# SARS-CoV-2 vaccines: Potential refinements through induction of mucosal and trained immunity

**DOI:** 10.1016/j.clinsp.2022.100057

**Published:** 2022-05-30

**Authors:** Amanda Izeli Portilho, Gabrielle Gimenes Lima, Elizabeth De Gaspari

**Affiliations:** a Departament of Immunology, Adolfo Lutz Institute, São Paulo, SP, Brazil; b Gradute Program Interunits in Biotechnology, Biomedical Sciences Institute, University of São Paulo, São Paulo, SP, Brazil

## Introduction

The presence of IgA antibodies at the beginning of infection by SARS-CoV-2 and in various locations, such as the blood, Bronchoalveolar Lavage (BAL), and saliva, demand attention, as well as its correlation with neutralizing antibodies. The early IgA appearance was recently described in very well-conducted research by Sterlin et al.^1^

With the emergence of SARS-CoV-2 variants, there are chances that COVID-19 vaccines will have to be further improved. The authors’ comprehension of immunity against SARS-CoV-2 should pave the way for the vaccine's refinement. Herein, the authors briefly discuss the issues related to this matter.

## Mucosal immunization

When the authors think about intranasal immunization, it comes to mind the story of the first Intranasal (IN) vaccination, documented in a Chinese medicine text, in the year 1742. It consisted of an attempt at immunization against variola. However, only in 2000 the first IN vaccine was approved for use in humans, in the United States: the FluMist® vaccine (AstraZeneca), against Influenza. The development of this vaccine led to investments in research related to the area.^2^

Despite several reports currently described in the literature on the advantages of intranasal vaccination, a worrying aspect of this immunization pathway was the association established between the vaccination with an inactivated influenza virus vaccine adjuvanted by the heat-labile toxin of *Escherichia coli* and Bell's Palsy, a facial nerve paralysis, in 2002‒2003.^3^ After this episode, it became clear that the IN delivery of vaccines should consider the anatomical proximity between the nasal cavity and the central nervous system. To increase their safety, such vaccines should use suitable adjuvants and transport systems, thus avoiding their deposition in the nervous system.^4^

Other points to discuss to the IN vaccines are related to the manufacturing and distribution, such as transport, adjuvant, vector, and dosage, not only the immune responses induced.^5^ Even in the current moment, where safe IN vaccines would benefit the control of COVID-19 pandemics, the authors have to be aware of its issues, especially the adjuvant use or delivery systems needed, as described above. As the authors know, it is not very easy for government agencies and non-profit organizations worldwide to support the commercialization of these technologies, thus leading to rapid mass vaccination.^6^ Thus, adjuvants and delivery systems can increase the cost of vaccines, making it even more difficult to expand vaccine coverage by IN formulations. Considering the moment that the world is going through, the scientific community cannot focus only on pointing out deficiencies, but also on putting effort to find solutions in a short time.

## Protective potential: variants of concern (VOCs) and avidity

Recently, the authors observed an increase in cases caused by the Omicron variant. Most vaccines use the Spike protein as antigen and several VOCs present mutations in this antigen to increase affinity towards ACE2 receptor.^5^^,^^7^ Therefore, the next SARS-CoV-2 vaccines should be refined to address this issue, protecting against VOCs and preventing new COVID-19 waves.

It is known that the longer the active infection, the higher the chance of VOCs arising. So, the activation of mucosal immunity would be interesting to limit the viral spread in the upper respiratory tract.^8^^,^^9^ With an earlier control of the infection, which could be provided by adequate mucosal immunity, it is less likely for SARS-CoV-2 to rearrange and mutate.

Another point is the functionality of antibodies. The importance of the affinity maturation of humoral response has been discussed.^7^ There is evidence that natural infection does not provide high avidity antibodies, which would fail in controlling the disease. On the other hand, vaccination has induced antibodies with higher avidity indexes, favoring the maturation of the immune response.^7^^,^^10^^,^^11^ All that points out that the avidity of antibodies must be observed for adequate vaccines.^7^

The avidity of mucosal IgA had not been studied extensively like IgG, making it harder to point out its role in preventing COVID-19.^12^ However, a study comparing the systemic IgA, IgM, and IgG response of COVID-19 patients to Spike pre-fusion epitope found increased avidity of recovered compared to deceased patients.^13^ Another investigation, which employed both serum and nasal wash samples verified a higher affinity of asymptomatic compared to symptomatic patients.^14^ Such results suggest that IgA avidity might improve the outcome of natural infection. The authors assume that the somatic mutations of mucosal plasma cells and IgA affinity are promising fields of study, which would support vaccine refinements as well.

## Vaccines beyond pathogen-specific prevention

Many reports described in the literature suggest that vaccines may have not only specific effects on the disease but on other infections. When Vaccinia, the first human vaccine, was introduced in the early 19th century, it was noticed an heterologous protection from various atopic diseases, such as measles, scarlet fever, and syphilis, aside from smallpox. When the Calmette-Guérin (BCG) bacillus vaccine was used in Sweden, mortality was almost 3 times lower among vaccinated children. As the main reduction in mortality occurs in childhood, it cannot be 100% explained by the prevention of tuberculosis, which killed mainly older children. Another relevant point to be investigated suggests stimulation with the nonspecific BCG vaccine another point to think about.^15^

There are not many studies in the literature on nonspecific immunity, which mostly suggest the engagement of B and T-cells and indicates a non-specific immune memory. There are also reports associating the participation of macrophages and Natural Killer (NK) cells. In addition, the participation of innate immunity involves epigenetic programming. It is safe to suppose that both innate and adaptive mechanisms take part in this cross-reactive immunity, given that the arms of the immune response are built to complement each other. This aspect, discussed as trained immunity, could generate a new understanding of immunological data related to vaccines and could thus impact resistance and general protection to the disease.^16^

The authors recall an example described in the literature that correlates immune system training related to the latency of the Herpes virus, which seems to protect against bacterial infection. That would happen through systemic activation of macrophages and production of IFN-γ, thus leading authors to suggest that herpes viruses are symbionts, rather than pathogens, in humans.^17^ It is important to think that the picture in which the innate immune system is characterized by adaptive features and can be trained to provide partial protection against infection independent of the classical T and B cell adaptive immunity deserves further studies.^18^

## Adequacy of SARS-CoV-2 vaccines

In the recent paper, Gaspar and De Gaspari^19^ observed Neutralizing antibodies to SARS-CoV-2 after IN administration of recombinant RBD. These experimental studies simulated the entry of the pathogen through the nasal route. The proposed platform using *Neisseria meningitidis* Outer Membrane Vesicles (OMVs) as adjuvant appears to be an alternative platform for a vaccine that stimulates different compartments of the immune system, culminating with the production of IgA antibodies soon after an IN dose of the antigen. Hence, outer membrane vesicles are technically easy and cheap to obtain, as an example of affordable adjuvants to improve vaccine manufacturing.

Another interesting point for investigation is the lymphatic drainages of the parental sites of immunization, due to the targeting of particular lymphoid inductive sites. Some of these sites may represent the crossroads between systemic and mucosal immunity.^20^ Thus, there is suggestive evidence that mucosal immunization can induce seric IgA because of B cells homing to the marginal zone of the spleen after activation at the mucosa.^21^ Considering the potential role of IgA to control COVID-19, these are aspects that are worthy of studying.

Although the authors managed to develop effective vaccines that have diminished the COVID-19 burden in different parts of the world,^22^ it is still relevant to evaluate different platforms including the use of different adjuvants, mucosa stimulation, and the key immunological compartments to be activated.^23^

## CRediT authorship contribution statement

**Amanda Izeli Portilho:** Writing – review & editing, Writing – original draft. **Gabrielle Gimenes Lima:** Writing – review & editing, Writing – original draft. **Elizabeth De Gaspari:** Conceptualization, Writing – review & editing, Writing – original draft.

## Conflicts of interest

The authors declare no conflicts of interest.
